# Effects of Quercetin and Citrulline on Nitric Oxide Metabolites and Antioxidant Biomarkers in Trained Cyclists

**DOI:** 10.3390/nu17020224

**Published:** 2025-01-09

**Authors:** Jennifer A. Kurtz, Rafaela G. Feresin, Jacob Grazer, Jeff Otis, Kathryn E. Wilson, J. Andrew Doyle, Kevin A. Zwetsloot

**Affiliations:** 1 Department of Public Health & Exercise Science, Appalachian State University, Boone, NC 28607, USA; zwetslootka@appstate.edu; 2 Department of Nutrition, Georgia State University, Atlanta, GA 30203, USA; rferesin@gsu.edu; 3 Department of Exercise Science and Sport Management, Kennesaw State University, Kennesaw, GA 30144, USA; 4 Department of Kinesiology & Health, Georgia State University, Atlanta, GA 30303, USAkwilson141@gsu.edu (K.E.W.); adoyle@gsu.edu (J.A.D.); 5 Department of Biology, Appalachian State University, Boone, NC 28607, USA

**Keywords:** endurance exercise, aerobic capacity, dietary supplements, oxidative stress, redox signaling

## Abstract

Background: Quercetin (QCT) and citrulline (CIT) have been independently associated with improved antioxidant capacity and nitric oxide (NO) production, potentially enhancing cardiovascular function and exercise performance. This study aimed to evaluate the combined and independent effects of QCT and CIT supplementation on NO metabolites and antioxidant biomarkers in 50 trained cyclists undergoing a 20 km cycling time trial (TT). Methods: In a randomized, double-blind, placebo-controlled design, forty-two male and eight female trained cyclists were assigned to QCT + CIT, QCT, CIT, or placebo (PL) groups. Supplements were consumed twice daily for 28 days. Biochemical assessments included NO metabolites (nitrate/nitrite), ferric reducing antioxidant power (FRAP), superoxide dismutase (SOD) activity, and antioxidant capacity, measured pre- and post-TT. Results: NO metabolites were significantly elevated post-supplementation (*p* = 0.03); however, no significant interaction effects were observed for NO metabolites, FRAP, SOD, or antioxidant capacity across the groups (*p* > 0.05). Post-hoc analyses revealed that QCT significantly reduced FRAP concentrations compared to PL (*p* = 0.01), while no significant changes in SOD or antioxidant capacity were found across any groups. Conclusions: These findings suggest that combined and independent QCT and CIT supplementation did not significantly improve these biomarkers, suggesting that baseline training adaptations, supplementation timing, and individual variability may influence the efficacy of these compounds in enhancing exercise performance and oxidative stress markers. The ergogenic efficacy of QCT + CIT on antioxidant-related markers remains inconclusive.

## 1. Introduction

Quercetin (QCT) is a polyphenol, more specifically, a parent flavonoid compound [1,2], that has been shown to have powerful antioxidant and anti-inflammatory properties [3,4]. QCT is commonly found in plant-based foods including apples, elderberries, citrus fruits, red wine, red onions, hot peppers, berries, kale, buckwheat tea, dark leafy greens, and capers [2,5,6]. One of the mechanisms by which QCT may exert its effects is through improving endothelium-dependent vasodilation [7]. In fact, QCT has been shown to improve nitric oxide (NO) levels and antioxidant status in rats [8]. Further, QCT acts in the glutathione pathway to enhance antioxidant capacity [9]. Taken together, QCT may prove to be a promising supplement for attenuating high levels of oxidative stress by enhancing the glutathione pathway and the activity of antioxidant enzymes, such as superoxide dismutase (SOD), as well as increasing antioxidant capacity, measured by ferric reducing antioxidant power (FRAP) [10,11]. However, to the best of our knowledge, research investigating QCT’s effects on NO metabolites or antioxidant capacity in trained individuals does not exist.

Citrulline (CIT) is a nonessential amino acid found in high concentrations in watermelon [12,13,14]. CIT is formed from arginine, an amino acid involved in several physiological roles including the urea cycle, protein synthesis, and the activity of NO synthase (NOS) enzymes, which also yields NO [14,15,16,17,18,19]. Chronic CIT supplementation increases NOS activity and NO production, decreases blood pressure, and may increase peripheral blood flow [13]. Elevated reactive oxygen species (ROS), especially superoxide, can reduce NO bioavailability by generating peroxynitrite, further promoting reductions in NO synthesis, leading to endothelial dysfunction and limited exercise performance [17,20]. Although blood flow enhancement is a proposed mechanism for the ergogenic potential of CIT, evidence supporting acute improvements in vasodilation, vascular conductance, and antioxidant potential after supplementation is scarce and inconsistent in trained athletes.

Previously, we demonstrated that QCT + CIT, QCT, and CIT supplements did not alter cycling 20 km time trial (TT) performance and average power, respiratory exchange ratio (RER), and rate of perceived exertion; however, QCT and CIT improved oxygen consumption (VO_2_) in trained cyclists [21]. Yet, several lines of research suggest that while they may not enhance performance individually, both QCT and CIT could improve aspects of cardiovascular health and NO metabolism. However, to date, no studies exist investigating the potential synergist roles of QCT + CIT on NO metabolites and antioxidant biomarkers in trained athletes after a maximal 20 km TT. To address these gaps in the current body of knowledge, we conducted a systematic investigation into the ergogenic effects of daily supplementation with QCT and CIT, both individually and in combination, over a 28-day period. This study specifically evaluated their impact on NO metabolite production, FRAP, SOD activity, and overall antioxidant capacity following a 20-kilometer TT. It was hypothesized [21] that daily supplementation with QCT, CIT, or QCT + CIT for 28 consecutive days would enhance NO metabolite production and improve FRAP, SOD, and antioxidant capacity compared to placebo (PL) after a cycling 20 km TT.

### Study Population

Participants included male and female cyclists who regularly competed in races (mountain, gravel, cross country, road, cyclocross). Cyclists were defined as Tier 2 of a six-tier framework to classify exercise/training and/or sports performance levels [22]. Tier 2 is defined as a trained, developed individual who identifies cycling as their main sport [23] and provides a sport-specific metric of training volume (average: 101.58 ± 64.36 to 285.92 ± 92.10 km/week and 11.16 ± 5.08 to 18.55 ± 7.41 h/week). The participants were recruited through local cycling teams and races and trained at least three times per week, currently trained with a stationary bike/trainer, trained at least three to five hours per week over the past three years [24,25], and trained with a purpose to compete [23,24,26]. All females were tested during their follicular phase (approximately day 0 to day 16, assuming a 30-day regular cycle [27]), which is when female sex hormone concentrations are relatively stable and most similar to other women [28]. Females with medically prescribed monophasic, biphasic, or triphasic oral contraceptives or who were perimenopausal [29] were excluded due to a possible decrease in peak oxygen uptake (volume of O_2peak_ per minute) [30]. Exclusion criteria included the following: greater than two days of resistance training per week; daily use of nonsteroidal anti-inflammatory drugs and/or use of anti-hypertensive medications; smoking (in the past six months); use of tetrahydrocannabinol (THC) or cannabidiol (CBD) products; diagnosis of chronic, systemic, or inflammatory diseases; pregnant women; females who have not had a period in the past 6 months (i.e., amenorrhea); documented intolerance to iron; and orthopedic injuries that may impact cycling performance testing. This study was approved by and carried out in accordance with the university’s Institutional Review Board for the protection of human subjects (IRB # H23189; Approval Date: 11 April 2022).

## 2. Materials and Methods

A randomized, placebo-controlled study design was employed. Participants visited the Applied Exercise Physiology laboratory on three separate occasions scheduled throughout the day (0700–1600) at the same time of day (±2 h), spanning a five-to-six-week period. Visits required participants to perform a 20 km cycling TT on three separate visits while examining average power, oxygen consumption (VO_2_), respiratory exchange ratio (RER), and rating of perceived exertion (RPE). Informed consent was obtained from all subjects involved in the study.

During the testing period, participants maintained their typical race training regimen but avoided strenuous exercise for at least 48 h prior to each visit, only participated in low-intensity exercise 24 h prior to each visit, and agreed to avoid the use of large-dose vitamin or mineral supplements (>100% of the recommended dietary allowances (RDAs)); nutritional supplements or ergogenic aids such as QCT, CIT, creatine, β-alanine, antioxidant medications, tocopherols, or flavonoid supplementation; herbs; and anti-inflammatory or hypertensive medications during the testing periods. Participants were asked to follow a diet moderate in carbohydrates and protein similar to what they would normally consume before a race prior to each visit. Participants completed a 24 h dietary recall before each visit to ensure diet replication for subsequent visits. These recalls were analyzed using an online food processor (version 11.1 ESHA Research) to standardize and verify dietary intake. Body mass was recorded before each visit.

### 2.1. Visit Descriptions

Visit 1 consisted of completing the informed consent process, health history/medical history questionnaire, 24 h dietary recall, injury history questionnaire, physical activity readiness questionnaire, dual energy X-ray absorptiometry (DEXA) body composition scan, measurement of height and weight, and a 20 km TT familiarization bout. Participants had their body composition (Lunar Prodigy encore: PR 510021), height, and body mass measured before the familiarization session. Visit 2 consisted of a baseline TT performance bout prior to a 28-day supplementation period. Visits 1 and 2 took place 72 h apart, avoiding strenuous exercise for at least 48 h before visit 2. After visit 2, participants were randomly assigned to one of four treatment groups (see below). Following the 28-day supplementation period, participants returned for visit 3 to perform the post-supplementation 20 km TT performance test. After completion of each 20 km TT, participants performed a self-selected 5-min cool-down session.

### 2.2. 20 km Time Trial Performance

Participants completed a 10-min warm-up session at a self-selected pace and intensity [31,32] before the 20 km TT. The TTs were performed on a Wahoo Core KICKR Smart Trainer (Wahoo Fitness, Atlanta, GA, USA) using the Zwift system virtual training app (Zwift Inc., Long Beach, CA, USA). The 20 km TT consisted of a reproducible, flat terrain course at a freely-selected pedaling cadence allowing for the collection of average power, as previously described [25,26,33,34,35]. The KICKR trainer was set to open test mode during the TT, allowing participants to change gears and intensity freely throughout. The participants were instructed to produce their maximal power output for the TT, adopt their personal pacing strategies [36,37,38], and to complete the total distance in the fastest time possible [33]. Participants were permitted to drink water as needed, select their own music, and listen to the same playlist during each visit.

### 2.3. Supplementation

Participants were randomly assigned, under double-blind conditions, to one of four groups: (1) QCT + CIT, (2) QCT, (3) CIT, or (4) PL. Powder-form supplements were dissolved in 16 oz of water. The composition of these powders were as follows: (1) QCT + CIT (500 mg of QCT dihydrate, 3.0 g of L-CIT, 3.5 g orange Crystal Light [Kraft Heinz, Chicago, IL, USA]), 2×/day; (2) QCT (500 mg of QCT dihydrate, 3.5 g orange Crystal Light), 2×/day; (3) CIT (3.0 g of L-CIT and 3.5 g orange Crystal Light), 2×/day; (4) PL (3.5 g orange Crystal Light). Supplements were consumed twice daily for 28 consecutive days [39,40,41] starting the day after visit 2. The QCT and CIT dosages were chosen based on previous research in which the supplements were observed to positively improve performance [39,40,41,42,43,44,45,46,47].

Participants were instructed to add the powdered supplements to 16 oz of water and consume them within 30 min of their first and last meals of each day. The zero-calorie orange-flavored Crystal Light package was added to mask any taste and ensure that participants remained blinded to their treatment group. The supplements were consumed in beverage form to enhance absorption [48,49]. Participants were required to add only filtered or bottled room-temperature water to the bottle; no other fluids were allowed in the mix. During the supplementation period, participants received a weekly phone call or text check-ins from a research team member and logged their physical activity, gastrointestinal (GI) symptoms, and supplement compliance. To ensure consistency, participants were required to track when they consumed the supplement on a daily supplement compliance dosing diary. If participants were not compliant and missed more than 10% (~5.6 supplement bags), a sensitivity analysis was performed to determine the extent to which non-compliance may or may not have influenced the primary outcome of NO metabolites and secondary outcomes of FRAP, SOD, and antioxidant capacity. Participants were required to track their physical activity on a compliance dosing diary, including their intensity (i.e., 6–20 RPE scale), mode, and duration, throughout the study. During the 4-week intervention, participants maintained their typical race training regimen while adhering to study protocols, which included avoiding strenuous exercise for at least 48 h and limiting activity to low-intensity exercise 24 h prior to each visit [21].

### 2.4. Blood Collection

Approximately 20 mL of blood was collected into two EDTA-treated BD Vacutainer^®^ tubes (BD Biosciences; Franklin Lakes, NJ, USA) from the antecubital vein pre-exercise and immediately post-exercise. The EDTA-treated tubes were gently inverted 8–10 times and centrifuged for 10 min at 3600 RPM at 4 °C. Plasma was aliquoted and stored at –20 °C for later analysis.

### 2.5. Biochemical Assessments

NO metabolites, FRAP, SOD, and antioxidant capacity, the ability to regulate free radicals produced by the body during metabolic processes [50], were measured using commercial assay kits, according to manufacturer instructions. The metabolic fate of NO involves its oxidation to its metabolites nitrate and nitrite [51]. These metabolites can improve cardiovascular function and biomarkers of oxidative stress by regulating blood flow and vascular tone, thereby influencing performance [52]. FRAP measures antioxidant potential in which ferric iron (Fe^3+^) is reduced to ferrous iron (Fe^2+^), thus reflecting the total antioxidant activity [53]. SOD is a metalloenzyme that plays a major role in antioxidant defense by catalyzing the dismutation of superoxide anion radicals into molecular oxygen (O_2_) and hydrogen peroxide (H_2_O_2_^)^ in the body [54].

NO metabolites were measured using Nitrate/Nitrite Colorimetric Assay Kit (cat. #780001), SOD using Superoxide Dismutase Assay Kit (cat. # 706002-96), and antioxidant capacity using Antioxidant Assay Kit (cat. # 709001-96) (all from Cayman Chemical, Ann Arbor, MI, USA). FRAP was measured using OxiSelect™ FRAP Assay Kit (cat. # STA-859, Cell Biolabs Inc., San Diego, CA, USA). Samples were assayed in duplicate. All samples from each subject were analyzed on the same plate. The intra- and inter-assay coefficient of variation (CV) for all blood measurements was <2%.

### 2.6. Statistical Analyses

A series of one-way ANOVAs were performed to examine differences among experimental conditions in continuous anthropometric, demographic, and performance variables at baseline (visit 1). Chi-squared analyses were performed to test for group differences in categorical variables at baseline. A repeated measures mixed model ANOVA (2 × 2 × 4; pre/post exercise bout, pre/post supplement, condition, respectively) was performed to assess mean differences in total NO metabolite production, FRAP, SOD, and antioxidant capacity. Effect sizes were expressed as partial eta squared (*η*^2^), and effect size thresholds were categorized and interpreted as small (*η_p_*^2^ = 0.01), medium (*η_p_*^2^ = 0.06), and large (*η_p_*^2^ = 0.14) [55]. In the event of a significant F-ratio, the model was decomposed using a series of between-group and repeated measures ANOVAs with Bonferroni correction. The variability of the NO metabolites was calculated using the CV. Percent change for each subject was calculated to assess the NO metabolites, FRAP, SOD, and antioxidant capacity concentrations from pre-supplementation to post-supplementation. Data are represented as mean ± SD. Significance was set at *p* < 0.05.

## 3. Results

Demographic characteristics are presented in Table 1, including a total of 50 male (*n* = 42) and female (*n* = 8) trained cyclists (ages 18–55) who regularly competed in category 1–3 cycling races across several disciplines, including mountain, gravel, cross country, road, and cyclocross. Baseline anthropometric measures for participants randomized to QCT + CIT (*n* = 11 males, 1 female), QCT (*n* = 9 males, 4 females), CIT (*n* = 11 males, 1 female), and PL (*n* = 11 males, 2 females) groups are summarized in Table 1. No significant differences were found for age, gender, ethnicity, or anthropometric measures at baseline (*p* > 0.05). There were no significant changes in menstrual cycles among women as all testing was performed during the participants’ follicular phase. Previously, we reported there were no significant differences in training volume or intensity between groups during the intervention, as confirmed by weekly activity logs and compliance checks [21]. Analyses of the 24 h dietary recall revealed no statistically significant within- and between-group differences. Minor gastrointestinal (GI) distress was reported by participants in all groups, including Q + CIT (*n* = 8), Q (*n* = 11), CIT (*n* = 10), and placebo (*n* = 6). Symptoms such as bloating, stomach heaviness, belching, abdominal pain, and difficulty with gas evacuation were noted. However, as only 29 participants completed all four weekly GI surveys, the data were insufficient to draw definitive conclusions about the impact of supplementation on GI tolerance.

Total weekly training distance ranged from 101.58 ± 64.36 to 285.92 ± 92.10 km. Cyclists’ total weekly time spent training ranged from 11.16 ± 5.08 to 18.55 ± 7.41 h. There were no differences in physical activity across supplement groups (*p* > 0.05). Further, there was a 92% supplement compliance rate across all the participants.

**Figure 1:**
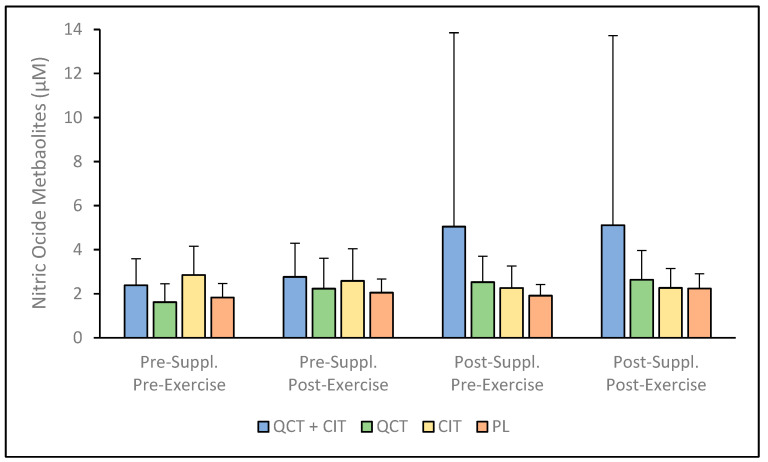
Plasma concentration of nitric oxide (NO) metabolites pre- to post-supplementation. Data are displayed as mean + SD. *n* = 47: QCT + CIT (*n* = 11), QCT (*n* = 13), CIT (*n* = 11), PL (*n* = 12).

There were no significant interactions for NO metabolites (ANOVA model [F (3, 46) = 2.21, *p* = 0.10]; see Supplementary Table S1); however, there was a significant main effect of time, revealing that NO metabolites were elevated after the supplementation period (visit 3) compared to before the supplementation period (visit 2), regardless of group, with a medium effect size [F (1, 46) = 5.52, *p* = 0.03, η^2^ = 0.11] (*p* < 0.01). We found no influences of visit [F (1, 46) = 1.35, *p* = 0.24] or supplement [F (3, 46) = 1.18, *p* = 0.33] on NO metabolites. The main effects of visit [F (1, 46) = 1.09, *p* = 0.30] and supplement [F (3, 46) = 1.06, *p* = 0.38] were not significant. The NO metabolite concentration was increased in the QCT group, albeit not significantly (Figure 1). The percent change in NO metabolites pre-to-post supplementation was observed to range from 15.21 to 1.25% [95% CI: 0.81–5.13] for QCT + CIT, 59.37 to 4.96% [95% CI: 0.60–0.68] for QCT, 6.43 to 4.22% [95% CI: 1.39–0.94] for CIT, and 17.61 to 19.56% [95% CI: 0.63–0.59] for PL.

**Figure 2:**
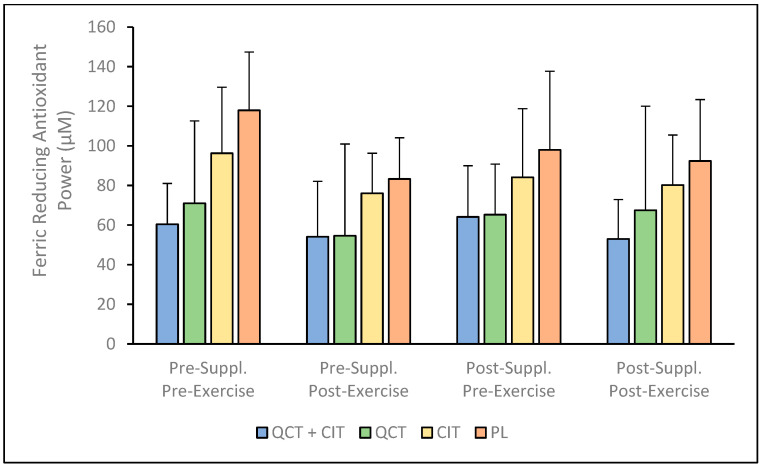
Ferric reducing antioxidant power (FRAP) concentration pre- to post-supplementation. Data are displayed as mean + SD. *n* = 40: QCT + CIT (*n* = 9), QCT (*n* = 11), CIT (*n* = 9), PL (*n* = 11).

There were no significant interactions for FRAP (ANOVA model [F (3, 36) = 0.88, *p* = 0.46]; Figure 2, see Supplementary Table S2). However, there were main effects of supplement [F (1, 36) = 3.21, *p* = 0.03, *η_p_*^2^ = 0.21] and visit [F (1, 36) = 8.01, *p* = 0.01, *η_p_*^2^ = 0.18]. Concomitantly, the effect sizes indicated a large effect according to Cohen’s criteria [55], suggesting that the timing of the visits contributed substantially to the changes observed in FRAP concentrations. Post-hoc pairwise comparisons using the Bonferroni correction revealed significant FRAP concentration differences between several groups. Specifically, QCT (57.92 ± 10.86 μM) was significantly different from PL (97.87 ± 9.83 μM) when comparing pre- to post-supplementation (−39.96 ± 14.65 μM; 95% CI [−69.66, −10.25], *p* = 0.01). This suggests that QCT had lower FRAP concentrations than the PL group overall. In contrast, the QCT + CIT (57.92 ± 10.86 μM) and CIT (84.57 ±9.83 μM) were not significantly different (−26.21 ± 15.36 μM, 95% CI [−57.37, 4.94], *p* = 0.10), suggesting there was no difference in FRAP concentrations from pre- to post-supplementation in these groups. However, QCT (64.57 ± 9.83 μM) was significantly different from PL (97.87 ± 9.83 μM) from pre- to post-supplementation (−33.30 ± 13.90 μM, 95% CI [−61.48, −5.12], *p* = 0.02), suggesting that QCT had lower FRAP concentrations compared to PL (Figure 2). The percent change in FRAP concentrations pre- to post-supplementation was observed to range from 4.81 to 15.00% (95% CI [15.86, 14.95]) for QCT+ CIT, 22.71 to 18.53% (95% CI [26.00–23.09]) for QCT, 13.13 to 4.10% (95% CI [26.84, 30.03]) for CIT, and 29.95 to 0.80% (95% CI [25.18, 35.40]) for PL.

**Figure 3:**
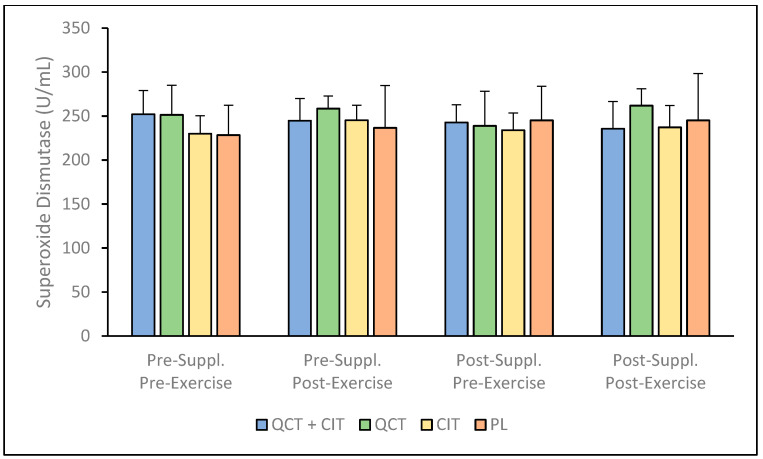
Superoxide dismutase (SOD) concentration pre-to-post supplementation. Data are displayed as mean + SD. *n* = 20: QCT + CIT (*n* = 6), QCT (*n* = 4), CIT (*n* = 6), PL (*n* = 4).

There were no observed significant differences for SOD in any of the analyses including the interaction [F (3, 16) = 0.88, *p* = 0.46] and the main effects for visit [F (1, 16) = 0.04, *p* = 0.95], time [F (1, 16) = 1.85, *p* = 0.19], and supplement [F (1, 16) = 0.38, *p* = 0.77] (Figure 3, see Supplementary Table S3). The percent change in SOD concentrations pre- to post-supplementation was observed to range from 2.48 to 3.05% (95% CI [20.91, 20.33]) for QCT + CIT, 3.83 to 11.23% (95% CI [23.54, 28.59]) for QCT, 7.05 to 1.27% (95% CI [15.04, 17.81]) for CIT, and 3.34 to 1.40% (95% CI [41.13, 45.90]) for PL.

**Figure 4:**
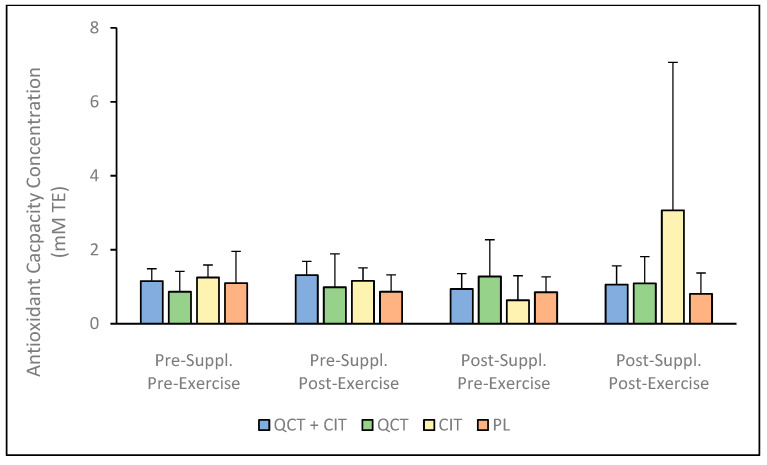
Antioxidant capacity concentration (AOC) pre-to-post supplementation. Data are displayed as mean + SD. *n* = 20: QCT + CIT (*n* = 6), QCT (*n* = 4), CIT (*n* = 6), PL (*n* = 4).

Lastly, for antioxidant capacity, there was no significant interaction [F (3, 16) = 1.23, *p* = 0.33] nor any significant main effects for visit [F (1, 16) = 0.14, *p* = 0.71], time [F (1, 16) = 0.80, *p* = 0.38], or supplement [F (1, 16) = 0.48, *p* = 0.70] (Figure 4, see Supplementary Table S4). Further, the percent change in antioxidant capacity concentrations from pre- to post-supplementation was observed to range from 17.54 to 30.22% (95% CI [0.28, 0.37]) for QCT + CIT, 1.80 to 15.97% for QCT (95% CI [0.73, 0.86]), 4.24 to 8.55% (95% CI [0.34, 2.34]) for CIT, and 27.44 to 15.13% (95% CI [0.65, 0.48]) for PL.

## 4. Discussion

This investigation into the biochemical effects of supplementing with a combination of QCT and CIT, as well as with QCT or CIT individually, revealed nuanced impacts. These findings highlight variations in their effectiveness and suggest potential underlying mechanisms influencing performance. To our knowledge, this study was the first to examine the effects of QCT + CIT, QCT, and CIT supplementation on these biomarkers after a 20 km maximal TT in trained males and females. Despite the lack of improvements in 20 km time trial performance across all groups, this study provides important insights into the physiological effects of QCT and CIT supplementation. Previously, our group reported that QCT and CIT combined supplementation did not elicit synergistic benefits on performance metrics, but QCT and CIT ingested individually resulted in improvements in VO_2_ (*p* = 0.05 and *p* = 0.04, respectively) [21]. These findings contribute to the growing body of research exploring the nuanced roles of these supplements in enhancing NO metabolite production and antioxidant biomarkers, which remain key areas of focus for optimizing endurance performance in trained athletes. The results reveal that QCT + CIT, QCT, and CIT supplementation do not improve plasma concentrations of NO metabolites, FRAP, SOD, or antioxidant capacity in trained cyclists (Figure 1, Figure 2, Figure 3 and Figure 4). There was a significant main effect of time on NO metabolite concentration which may be explained by the large within-individual or biological variation between visits. It is possible there may be a sex effect and/or an effect of supplementation duration on NO mediators or related physiological adaptations [56]. Future studies should consider these effects to further understand the impact of sex and/or supplementation length on NO metabolites. Consistent with previous research, the significant variability in the concentration of NO metabolites observed in the QCT + CIT group may be attributed to considerable differences in how individuals process plasma nitrate and nitrite before and after supplementation [6]. While the nitrate–nitrite–NO pathway may influence muscle function and exercise performance, research is limited in humans.

Furthermore, no significant interaction effects were observed for NO metabolites, which aligns with previous research indicating that NO markers did not enhance performance in trained individuals [57,58]. However, supplements targeted to improve NO metabolites and their effects on trained athletes’ aerobic performance are limited. One possible explanation is the low bioavailability or metabolism of L-arginine, influencing NO and its metabolites [57]. Exhaustive exercise increases arginase enzyme activity, reducing L-arginine availability [59]. Additionally, high lysine concentrations in the diet may compete with L-arginine for cellular entry, possibly preventing an increase in, or at least bioavailability of, NO metabolites [57,60]. This competition may exacerbate the negative effects of high oxidative stress experienced during maximal exercise.

High oxidative stress can impair mitochondrial synthesis in skeletal muscle, reducing ATP production and exercise performance [61]. Studies have demonstrated that QCT can enhance antioxidant capacity and SOD activity [5,62]. McAnulty et al. [41] investigated the chronic effects of QCT on exercise-induced oxidative damage using FRAP and Trolox equivalent antioxidant capacity markers. While exercise elevated these markers, no significant differences were observed between groups. Similarly, in our study, the 20 km TT did not elicit a significant effect on oxidative markers. However, our results suggest that antioxidant capacity, measured by FRAP, was maintained in the QCT + CIT, QCT, and CIT groups. This aligns with previous findings which indicate that QCT supplementation can stabilize or increase FRAP during oxidative stress [63]. Previously, CIT–malate supplementation reduced muscle fatigue and increased NO production, which may also help to maintain FRAP levels [64]. Combined antioxidant supplementation may more effectively mitigate oxidative stress compared to single compounds, helping to maintain or increase FRAP levels during or after exercise [65]. The decrease in FRAP in the PL group likely reflects exercise-induced oxidative stress, while the QCT + CIT, QCT, and CIT groups maintained their FRAP levels, likely due to the antioxidant properties and synergistic effects of these supplements. The antioxidant capabilities of QCT are partly due to its phenolic structure, which enables free radical scavenging; however, research, including recent findings [21], highlights that polyphenols also exert their effects by interacting with specific molecular targets. Similar to what has been previously reported, the absorption and bioavailability of QCT likely varied among our participants [66]. Granado-Serrano et al. [67] found that QCT enhances cellular antioxidant capacity by activating the p38MAPK pathway and increasing intracellular glutathione levels; however, these effects may have already been present in our trained population. This contrasts with findings in untrained individuals, where CIT supplementation notably increased SOD levels post-exercise, suggesting that the response to these supplements can differ based on training status and individual variability [68]. In contrast, we did not observe significant differences in oxidative stress markers post-exercise in our study, possibly due to differences in protocol and population [69]. The antioxidant effects of CIT seem to depend on the nature of the oxidative stress and its concentration in vivo, with higher concentrations potentially being less effective [16]. We may not have captured the optimal peak of antioxidant enzyme function, which is reported to occur around 2 h post-endurance exercise [70]. Moreover, chronic training in our subjects likely enhanced their baseline antioxidant enzyme activity, potentially overshadowing the effects of QCT + CIT, QCT, and CIT supplementation on NO metabolites and antioxidant capacity. Further, recent studies indicated that excessive intake of antioxidants, such as vitamins C and E, may impair exercise-induced physiological adaptations by disrupting redox signaling pathways essential for muscle adaptation and performance improvement [71]. Additionally, research on antioxidant supplements and endurance exercise suggests that while antioxidants are commonly consumed to minimize exercise-induced oxidative stress, their efficacy in enhancing performance is not well-supported [72], and their use may even be detrimental. It is possible that QCT + CIT, QCT, and CIT supplementation may have mitigated inflammatory responses, which could potentially have impeded the beneficial effects on TT performance. Trained individuals with higher baseline FRAP, SOD, and antioxidant capacity are at lower risk of exercise-induced oxidative stress compared to untrained individuals with lower baseline antioxidant enzyme activity. Given these findings, it is crucial to approach antioxidant supplementation with caution, particularly concerning high doses, as it may counteract desired training adaptations and performance improvements in athletes.

The timing and bioavailability of supplementation likely influenced our results, as the peak absorption of NO metabolites could have decreased by the start of the 20 km TT. QCT has a half-life of 11–28 h, with peak concentrations occurring 1–3 h post-ingestion [73,74,75,76], while CIT has a shorter half-life of approximately 60 min [77]. A potential limitation of this study is the variability in the timing of supplement consumption, with some cyclists consuming their last dose on the day of the time trial and others 24 h prior. This inconsistency may have affected our ability to capture the supplements’ optimal absorption and peak concentration window. Future research should focus on investigating the acute effects of QCT + CIT on NO metabolites within this optimal window to better detect possible supplementation effects [78,79,80]. The beneficial effects of QCT and CIT in humans largely depend on their bioavailability [75]. The bioavailability of QCT is influenced by co-ingestion with nutrients, gut microbiota, and glycosides [81], further highlighting the need for precise timing in supplementation studies in trained individuals [78,79,80]. However, while the effects of nitrate supplementation on endurance exercise remain inconclusive, evidence suggests that it may improve time to exhaustion, further underscoring the need for targeted approaches to optimize supplementation benefits [82].

A limitation of the current study is that the Q, CIT, and placebo powders were not analyzed for nutrient composition and therefore their nutritional information was derived from bulk supplements. To allow for an accurate comparison of the nutritional content of the supplements being studied, future studies need to examine the overall nutrient density scores and quality of the supplements to avoid associated risks and obtain the greatest possible benefits from their consumption [83]. For future research, it is suggested to test the quality of these supplements, analyze their content, and verify if there are any differences between the information provided and the actual content, such as the presence of other undeclared ingredients. Moreover, fluid intake was not standardized prior to laboratory visits, nor were hydration levels assessed, which may have influenced the cardiovascular measurements. Future studies need to standardize and/or track fluid intake and hydration levels pre-, during, and post-exercise [84,85].

Further, future studies should control for dietary factors to account for metabolic variability and the effects of low doses of bioactive supplements [86]. It is possible that the cyclists’ elevated baseline levels of nitrite, nitrate, and antioxidant enzymes, potentially influenced by their diet and training adaptations, may have blunted their response to supplementation [86]. Additionally, trained athletes might not benefit from further supplementation due to adequate dietary intake and training-induced adaptations [87,88,89]. Future research should address the biological variability of these biomarkers and establish normative thresholds for trained athletes. Given the limited research available, further studies are needed to explore how factors such as training status, age, sex, and supplementation duration influence the effects of nitrate supplementation on exercise performance [82].

## 5. Conclusions

Previous findings from our group indicated that QCT + CIT, QCT, and CIT supplementation did not affect 20 km cycling TT performance, average power, respiratory exchange ratio (RER), or perceived exertion. However, QCT and CIT improved oxygen consumption (VO_2_) in trained cyclists [21]. While the data do not offer definitive conclusions regarding the effects of QCT and CIT on NO metabolites and antioxidant biomarkers post-exercise, further research is warranted to investigate localized changes in additional biomarkers and mediators during recovery. Future studies should also examine the impact of extended supplementation duration, prolonged exercise, and baseline dietary antioxidant or NO metabolite levels on the efficacy of QCT or QCT combined with other polyphenols.

## Figures and Tables

**Table 1:** Cyclist demographic characteristics at baseline familiarization testing, visit 1 [21].

	QCT + CIT (*n* = 12)	QCT (*n* = 13)	CIT (*n* = 12)	PL (*n* = 13)
Age (yr)	33 ± 1	35 ± 1	37 ± 1	37 ± 1
Height (cm)	176 ± 2	173 ± 1	178 ± 1	177 ± 1
Body Mass (kg)	78.2 ± 1.8	74.6 ± 2.0	79.8 ± 1.2	77.4 ± 1.1
Lean Tissue (kg or %)	59.4 ± 1.1	55.8 ± 1.5	59.0 ± 1.1	59.0 ± 0.9
Body Fat (%)	20.9 ± 0.8	22.9 ± 0.9	23.3 ± 1.0	20.9 ± 1.0
Total Weekly Cycling Volume (AU)	1416 ± 54	1447 ± 62	1347 ± 120	1619 ± 87
Sex, n (%)				
Females	1 (2)	4 (8)	1 (2)	2 (4)
Males	11 (22)	9 (18)	11 (22)	11 (22)
Average VO_2_ (mL/kg/min)	40.01 ± 6.72	40.50 ± 7.18 *	38.56 ± 5.66 *	40.56 ± 7.43
Time Trial Performance (minutes)	30.27 ± 2.35	29.96 ± 2.36	30.93 ± 2.69	30.82 ± 3.19

Data are presented as mean ± SD. VAT = visceral adipose tissue. Total weekly cycling volume is expressed as arbitrary units and is calculated as the rating of perceived exertion * total daily minutes/total exercised days out of 28. VO_2_ = oxygen consumption at baseline, visit 1.

## Data Availability

The data supporting the findings of this study are not publicly available due to privacy reasons. However, they can be made available from the corresponding author upon reasonable request.

## Supplementary Materials

The following supporting information can be downloaded at: https://www.mdpi.com/article/10.3390/nu17020224/s1, Table S1. Mixed model repeated measures within- and between-subjects ANOVA for NO metabolites; Table S2. Mixed model repeated measures within- and between-subjects ANOVA for FRAP, *n* = 40; Table S3. Mixed model repeated measures within- and between-subjects ANOVA for superoxide dismutase, *n* = 20; Table S4: Mixed model repeated measures within- and between-subjects ANOVA for antioxidant capacity, *n* = 20.
