# Supplementary material for: Effects of Quercetin and Citrulline on Nitric Oxide Metabolites and Antioxidant Biomarkers in Trained Cyclists
Source: Nutrients. 2025 Jan 9;17(2):224. doi: 10.3390/nu17020224 (PMC11767657; doi:10.3390/nu17020224)
Supplement: Supplementary file 1 [file nutrients-17-00224-s001.zip › nutrients-3375729-supplementary.pdf]

Supplementary Materials

Supplementary Table S1. Mixed model repeated measures within-and-between subjects ANOVA for nitric oxide (NO) metabolites.

**Variable**	***F***	***df***	***p***	η_p^2	***Observed Power***
**NO Metabolites**					
**Visit*Time*Supplement**	2.21	3.00	0.10	0.13	0.52
**Visit*Time**	0.82	1.00	0.37	0.02	0.14
**Visit*Supplement**	1.08	3.00	0.37	0.07	0.27
**Time*Supplement**	1.83	3.00	0.16	0.11	0.44
**Visit**	1.41	1.00	0.24	0.03	0.21
**Time**	5.22	1.00	0.03*	0.11	0.61
**Supplement**	1.18	3.00	0.33	0.08	0.29

Supplementary Table S2. Mixed model repeated measures within-and-between subjects ANOVA for ferric reducing antioxidant power (FRAP).

**Variable**	***F***	***df***	***p***	η_p^2	***Observed Power***
**FRAP**					
**Visit*Time*Supplement**	0.88	3.00	0.46	0.07	0.22
**Visit*Time**	3.91	1.00	0.06	0.10	0.49
**Visit*Supplement**	0.51	3.00	0.68	0.18	0.14
**Time*Supplement**	0.10	3.00	0.96	0.01	0.07
**Visit**	8.01	1.00	0.01**	0.18	0.79
**Time**	0.03	1.00	0.87	0.00	0.05
**Supplement**	3.21	3.00	0.03*	0.21	0.69

Supplementary Table S3. Mixed model repeated measures within-and-between subjects ANOVA for superoxide dismutase (SOD).

**Variable**	***F***	***df***	***p***	η_p^2	***Observed Power***
**SOD**					
**Visit*Time*Supplement**	0.88	3.00	0.47	0.14	0.20
**Visit*Time**	0.02	1.00	0.97	0.00	0.05
**Visit*Supplement**	0.70	3.00	0.57	0.12	0.17
**Time*Supplement**	1.39	3.00	0.28	0.21	0.30
**Visit**	0.04	1.00	0.95	0.00	0.05
**Time**	1.85	1.00	0.19	0.10	0.25
**Supplement**	0.38	3.00	0.77	0.07	0.11

Supplementary Table S4. Mixed model repeated measures within-and-between subjects ANOVA for antioxidant capacity.

**Variable**	***F***	***df***	***p***	η_p^2	***Observed Power***
**Antioxidant capacity**					
**Visit*Time*Supplement**	1.23	3.00	0.33	0.19	0.27
**Visit*Time**	1.67	1.00	0.36	0.05	0.14
**Visit*Supplement**	0.42	3.00	0.74	0.07	0.12
**Time*Supplement**	0.99	3.00	0.42	0.16	0.22
**Visit**	0.14	1.00	0.71	0.01	0.07
**Time**	0.80	1.00	0.38	0.05	0.13
**Supplement**	0.48	3.00	0.70	0.08	0.13
